# Elecampane (*Inula helenium*) Root Extract and Its Major Sesquiterpene Lactone, Alantolactone, Inhibit Adipogenesis of 3T3-L1 Preadipocytes

**DOI:** 10.3390/molecules27154765

**Published:** 2022-07-25

**Authors:** Yeon-Seop Jung, Yun-Jeong Jeong, Joung-Hee Kim, Chang-Hwan Jeon, Syng-Ook Lee

**Affiliations:** 1 Department of Food Science and Technology, Keimyung University, Daegu 42601, Korea; jys86170@dgmif.re.kr (Y.-S.J.); jch6203@naver.com (C.-H.J.); 2 Preclinical Research Center, Daegu-Gyeongbuk Medical Innovation Foundation (DGMIF), Daegu 41061, Korea; 3 Department of Medicine, Research Institute of Biomedical Engineering, Catholic University of Daegu School of Medicine, Daegu 42472, Korea; hinaky012@nate.com; 4 Laboratory of Molecular Toxicology, College of Pharmacy, Pusan National University, Pusan 46241, Korea; k9j1h@naver.com

**Keywords:** adipogenesis, Nur77, AMPKα, alantolactone, *Inula helenium*, elecampane

## Abstract

Recent studies have shown that Nur77 and AMPKα play an important role in regulating adipogenesis and isoalantolactone (ISO) dual-targeting AMPKα and Nur77 inhibits adipogenesis. In this study, we hypothesized that *Inula helenium* (elecampane) root extract (IHE), which contains two sesquiterpene lactones, alantolactone (ALA) and ISO, as major compounds, might inhibit adipogenesis. Here, we found that ALA and IHE simultaneously target AMPKα and Nur77 and inhibited adipogenic differentiation of 3T3-L1 cells, accompanied by the decreased expression of adipocyte markers. Further mechanistic studies demonstrated that IHE shares similar mechanisms of action with ISO that reduce mitotic clonal expansion during the early phase of adipogenic differentiation and decrease expression of cell cycle regulators. These results suggest that IHE inhibits adipogenesis, in part, through co-regulation of AMPKα and Nur77, and has potential as a therapeutic option for obesity and related metabolic dysfunction.

## 1. Introduction

The orphan nuclear receptor Nur77 is known to be expressed transiently in the early phase of adipogenic differentiation and stimulates the differentiation of 3T3-L1 cells by regulating mitotic clonal expansion (MCE) [1,2]. AMP-activated protein kinase α (AMPKα) is also known to play crucial roles in the early phase of adipogenesis [2,3]. Moreover, a recent study in this laboratory confirmed that the simultaneous regulation of AMPKα and Nur77 strongly inhibits adipogenesis, suggesting that compounds that simultaneously regulate AMPKα and Nur77 may be a new class of anti-adipogenic agents [2]. For example, isoalantolactone (ISO), a sesquiterpene lactone dual targeting AMPKα and Nur77, significantly inhibited adipogenesis in 3T3-L1 cells by decreasing MCE and reduced body fat mass in high-fat diet-induced obese mice [2].

*Inula helenium*, a member of the composite family, has long been grown in Southern Europe and Asia and has been used as herbal medicine to improve respiratory and digestive diseases. In addition, *Inula helenium* root extracts are rich in sesquiterpene lactones, mainly alantolactone (ALA) and ISO [4,5], and several studies have demonstrated the potential anticancer properties of these two compounds [6,7]. However, studies investigating the positive health effects of *Inula helenium* are limited, and underlying mechanisms for the antiadipogenic effects of ALA and *Inula helenium* are still largely unknown.

We thus hypothesized that *Inula helenium* root extract (IHE), which contains a large amount of the sesquiterpene lactones, may inhibit adipogenesis of 3T3-L1 cells. In the present study, we show that both ALA and IHE inhibit adipogenesis of 3T3-L1 cells in part via inhibition of the MCE-mediated early phase of adipogenesis, a similar mechanism to ISO.

## 2. Material and Methods

### 2.1. Antibodies, Reagents, Plasmids, and Luciferase Reporter Assay

Cyclin D1 and cyclin A antibodies were obtained from Santa Cruz Biotechnology (Santa Cruz, CA, USA) and all other antibodies were obtained from Cell Signaling Technology (Beverly, MA, USA). ALA and ISO were purchased from Toronto Research Chemicals (Toronto, ON, Canada). All reagents for reporter assay were obtained from Promega (Madison, WI, USA), and the plasmids were constructed as described previously [8,9]. For transfection with a reporter assay, cells (3 × 10^4^ cells/well) were plated onto 48-well plates, grown overnight, and then cotransfected with 25 ng of each reporter plasmid and the β-galactosidase reporter construct (5 ng) for 5 h using Lipofectamine 2000 reagent (Invitrogen, Carlsbad, CA, USA), following manufacturer’s protocol. Luciferase and β-galactosidase activities were determined, and luciferase activity was normalized with β-galactosidase activity.

### 2.2. Preparation of IHE Extracts

*Inula helenium* root was purchased from Starwest Botanicals (Sacramento, CA, USA). The dried root was extracted twice with 30% and 70% ethanol for 24 h at 25 ± 2 °C. The filtered extract was then concentrated under reduced pressure and lyophilized.

### 2.3. High-Performance Liquid Chromatography (HPLC) Analysis

HPLC analysis was carried out as described previously [10], with some modifications. A Shimadzu 06,959 series HPLC system (Shimadzu, Kyoto, Japan) and AkzoNobel KR100-5C18 column (AkzoNobel, Amsterdam, The Netherlands; pore size, 3.5 μm; 4.6 × 250 mm) were used for the analysis, and LC solution software (version 1.24, Kyoto, Japan) was used for data analysis. The mobile phases were formic acid aqueous (solvent A; 0.1%, *v*/*v*) and acetonitrile (solvent B). The gradient flow was as follows: 0–10 min 13% B, 10–12 min 13–50% B, and 12–40 min 50% B. The column was maintained at 30 °C, and the analysis was performed at a 1 mL/min flow rate with UV detection at 220 nm.

### 2.4. Cell Viability Assay and Oil Red O Staining

3T3-L1 cells were obtained from the American Type Culture Collection (Rockville, MD, USA) and maintained in a humidified 5% CO_2_ atmosphere at 37 °C with Dulbecco’s Modified Eagle’s Medium containing 10% bovine calf serum and penicillin/streptomycin (100 units/mL). Adipogenic differentiation of 3T3-L1 cells was induced by treating a differentiation cocktail (0.5 mmol/L 3-isobutyl-1-methylxanthine, 1 μmol/L dexamethasone, and 5 μg/mL insulin; MDI) for 3 days. The medium was then replaced every other day with a fresh growth medium containing insulin only. Differentiation was confirmed by noting lipid droplet accumulations, and Oil Red O staining and cell viability assay were undertaken as previously described [2].

### 2.5. Western Blot, Quantitative Real-Time PCR, and Bromodeoxyuridine (BrdU) Cell Proliferation Assays

Western blot and quantitative real-time PCR (qPCR) analyses were carried out as described previously [11], and Supplementary Table S1 shows the primer’s sequences. Cell proliferation induced by MDI was analyzed by BrdU incorporation assay. Briefly, 3T3-L1 cells were seeded and grown for at least two days post-confluency in a 96-well plate, and cells were then treated with MDI in the presence or absence of ISO for 12 h. Then, BrdU solution was added to the medium for 2 h and BrdU incorporation into the DNA was measured using the BrdU Cell Proliferation Assay Kit (BioVision, Milpitas, CA, USA) following the manufacturer’s protocol.

### 2.6. Statistical Analyses

Statistical significance of differences between groups was determined by either one-way ANOVA with Duncan’s multiple range test (SPSS, version 23.0, SPSS Inc., Chicago, IL, USA) or a *t*-test using Sigma Plot 10.0 (Systat Software Inc., San Jose, CA, USA). Results were presented as means ± SEM (*n* ≥ 3), and *p*-values less than 0.05 were considered statistically significant.

## 3. Results

### 3.1. ALA Targets Both Nur77 and AMPKα and Inhibits MDI-Induced Adipogenesis in 3T3-L1 Cells

**Figure 1:**
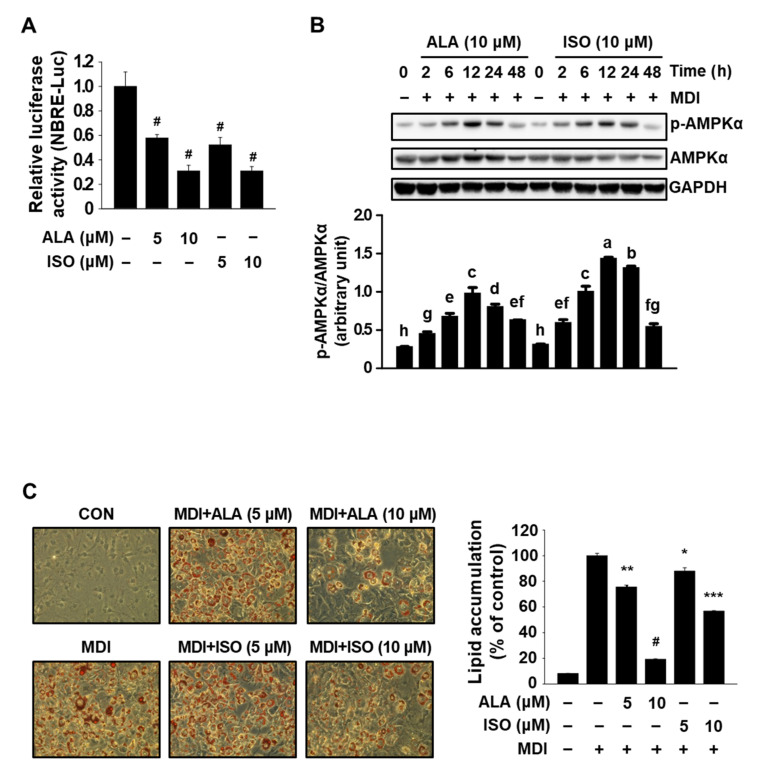
ALA and ISO simultaneously regulate Nur77 and AMPKα and inhibit the MDI-induced adipogenic differentiation of 3T3-L1 cells. (**A**) MiaPaCa-2 cells were cotransfected with Flag-Nur77 (12.5 ng) and NBRE-Luc (25 ng) for 4 h and then treated with ISO and ALA for 18 h. Luciferase activity (relative to β-galactosidase) was then determined; ^#^ *p* < 0.001 vs. DMSO + Nur77. (**B**) 3T3-L1 cells were treated with each compound in the presence of MDI for the indicated time period and whole cell lysates were analyzed by Western blot analysis. Different letters indicate a significant difference at *p* < 0.05. (**C**) 3T3-L1 cells were treated with each compound in the presence of MDI for 7 days and intracellular lipid accumulation was measured; * *p* < 0.05, ** *p* < 0.01, *** *p* < 0.005, and ^#^ *p* < 0.001 vs. MDI alone.

Prior to the investigation into the antiadipogenic effect of IHE, we first determined the effects of ALA, a positional isomer of ISO, on Nur77, AMPKα, and MDI-induced adipogenesis of 3T3-L1 cells. To test the inhibitory effect of ALA on Nur77, ALA was treated in cells transfected with a luciferase construct harboring Nur77-binding response elements (NBRE-luc). ALA (5 and 10 μM) was found to decrease the luciferase activity of NBRE-luc to levels similar to ISO in cells transfected with NBRE-luc (Figure 1A). Similar to ISO, ALA also induced the activation (phosphorylation) of AMPKα in MDI-treated 3T3-L1 cells (Figure 1B), indicating that both ALA and ISO simultaneously target Nur77 and AMPKα. It was also observed that phosphorylation of AMPKα started to occur at 2 h, reached a maximum at 12 h, and lasted for at least 24 h in MDI-treated 3T3-L1 cells.

Treatment with ISO has been shown to disturb MDI-induced adipogenesis in 3T3-L1 preadipocytes [2], and thus, the antiadipogenic effect of ALA was determined in MDI-treated 3T3-L1 cells. Growth-arrested 3T3-L1 cells were treated with MDI in the presence of 5 and 10 μM of ALA or ISO for 7 days, and the amount of intracellular lipid accumulation was compared by means of an Oil Red O staining. Over 70–80% of the cells were differentiated into typical adipocytes after 7 days of MDI treatment and the cells cotreated with ALA at both concentrations showed a low level of intracellular lipid accumulation (*p* < 0.05) comparable to ISO-treated cells (Figure 1C). When further measuring the cell viability, similar to ISO which is known to decrease cell proliferation by inhibiting MDI-induced MCE in growth-arrested 3T3-L1 cells [2], ALA treatment for 7 days also decreased MDI-induced cell proliferation (Supplementary Figure S1), suggesting that ALA inhibits MDI-induced adipogenesis in 3T3-L1 cells, in part, through similar mechanisms as ISO, such as the simultaneous regulation of Nur77 and AMPKα.

### 3.2. Quantitative Analysis of ALA and ISO in IHE

**Figure 2:**
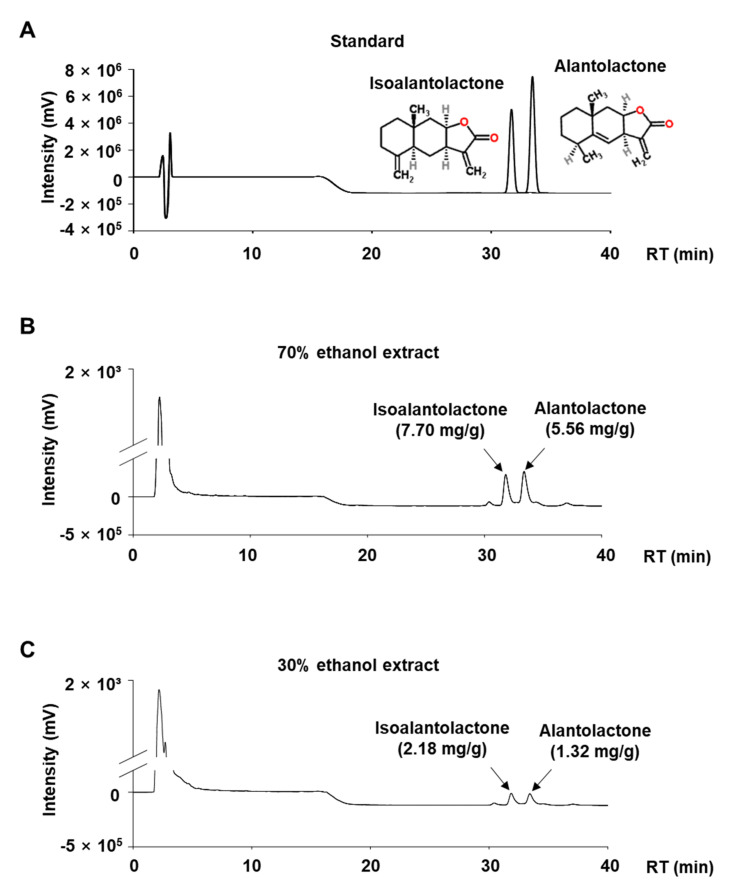
HPLC chromatograms of ISO and ALA in IHE. HPLC chromatograms of isoalantolactone and alantolactone standards (**A**), 70% ethanol extracts (**B**), and 30% ethanol extracts (**C**) of *Inula helenium* recorded at 220 nm.

We performed a quantitative analysis of ALA and ISO in IHE using HPLC analysis. As previously reported [4,5], we identified two sesquiterpene lactones, ALA and ISO, as the major marker compounds in IHE (Figure 2). Their retention times were 31 and 33 min, respectively. In addition, a 70% ethanol extract contains larger amounts of ALA (5.56 mg/g of extract) and ISO (7.7 mg/g of extract), compared to a 30% ethanol extract which contains 1.32 mg/g of extract of ALA and 2.18 mg/g of extract of ISO. Extraction yields of ALA and ISO in IH have been reported to increase in proportion to ethanol concentration and reach a maximum ratio in 80% extract, while slightly decreasing in 100% ethanol extract [12]. Therefore, the 70% ethanol extract (IHE) with higher ALA and ISO content was used for all further studies.

### 3.3. IHE Simultaneously Regulates Nur77 and AMPKα and Inhibits Adipogenesis in MDI-Treated 3T3-L1 Cells

**Figure 3:**
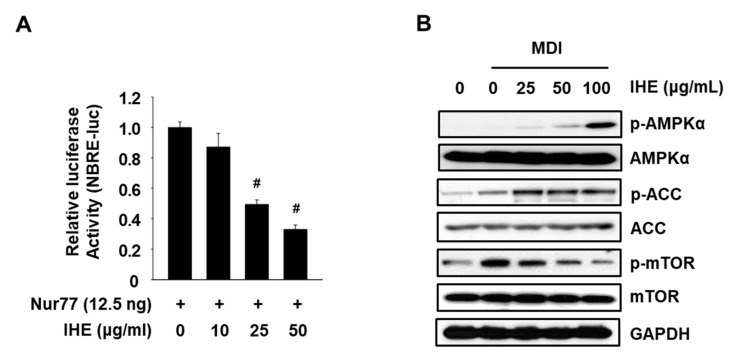
IHE simultaneously targets Nur77 and AMPKα. (**A**) NBRE-Luc (25 ng) was cotransfected with Flag-Nur77 (12.5 ng) into MiaPaCa-2 cells for 4 h and treated with IHE for 18 h. (**B**) 3T3-L1 cells were treated with IHE in the presence of MDI for 12 h. AMPKα expression was then determined by Western blot analysis; ^#^ *p* < 0.001 vs. DMSO control.

**Figure 4:**
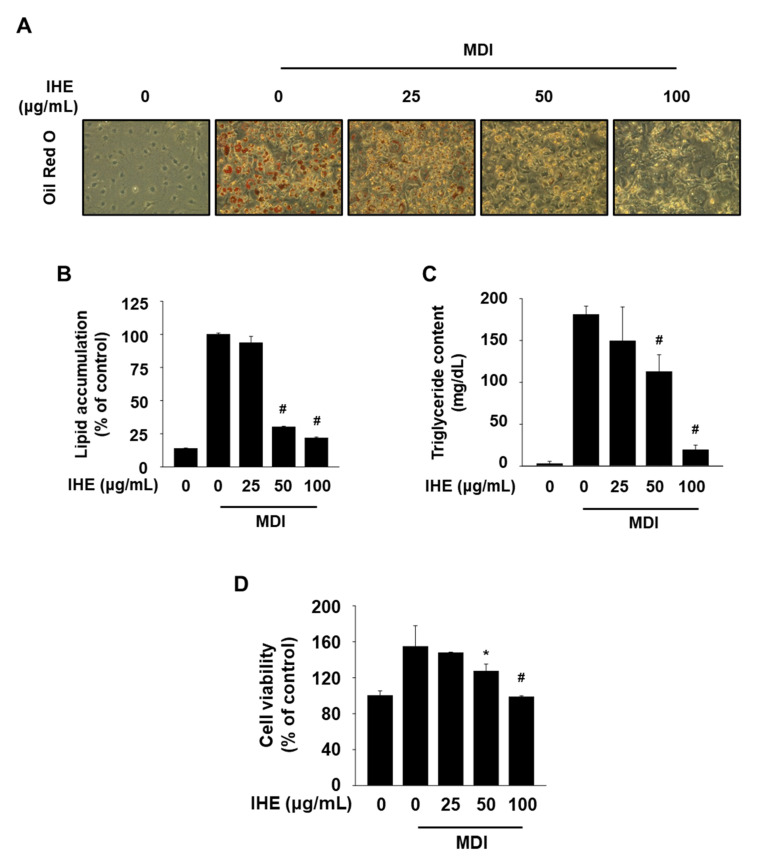
Effect of IHE on MDI-induced adipogenic differentiation of 3T3-L1 cells. (**A**–**C**) Cells were treated with IHE for 7 days in the presence of MDI and then intracellular lipid accumulation and TG content were measured. (**D**) Cell viability was measured at 7 days by an MTT assay; * *p* < 0.05, ^#^ *p* < 0.001 vs. MDI alone.

To examine whether IHE influences Nur77 and AMPKα, we performed Western blot and the promoter assay using transiently transfected cells with NBRE-luc. As shown in Figure 3A,B, IHE at 25 and 50 μg/mL significantly decreased the NBRE reporter gene activity, and treatment with IHE (25–100 μg/mL) also activated AMPKα and ACC and inhibited phosphorylation of the mammalian target of rapamycin (mTOR) which is downregulated by AMPKα in MDI-treated 3T3-L1 cells. Next, to investigate the effect of IHE on adipogenesis, the level of intracellular lipid accumulation in 3T3-L1 cells was compared after treatment with MDI in the presence of 25, 50, and 100 μg/mL of IHE for 7 days. The cells were well differentiated 7 days after the treatment with MDI alone, however, treatment with IHE at higher concentrations (50 and 100 μg/mL) significantly inhibited the intracellular lipid accumulation and TG content (Figure 4A–C). Furthermore, the cells treated with IHE for 7 days showed reduced cell viability compared to the MDI control cells (Figure 4D), suggesting that the antiadipogenic effect of IHE may also be related to MCE inhibition, similar to ALA and ISO.

**Figure 5:**
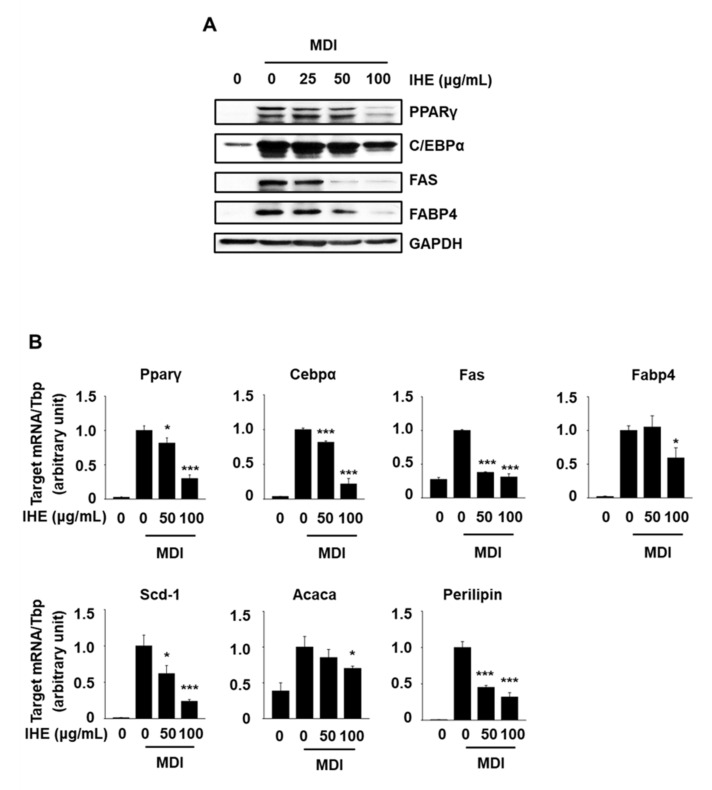
Effects of IHE on the expression of adipogenic transcription factors and their target genes. (**A**,**B**) Cells were treated with IHE for 6 days and protein and mRNA levels were determined by Western blot and qPCR analyses, respectively; * *p* < 0.05, *** *p* < 0.001 vs. MDI alone.

We used Western blot and qPCR analyses to further determine the inhibitory effects of IHE on the expression of adipocyte markers in MDI-treated 3T3-L1 cells. Protein expression of C/EBPα, FABP4, FAS, and PPARγ was increased in differentiated cells on day 6 compared to levels in 3T3-L1 preadipocytes (Figure 5A). However, IHE treatment at 50 and 100 μg/mL significantly reduced the expression of all these proteins during adipogenesis, whereas IHE treatment at 25 μg/mL showed no effect. Moreover, mRNA expression levels of Cebpα and Pparγ and their downstream target genes (Acaca, Fabp4, Fas, perilipin, and Scd1) were increased during adipogenesis. However, IHE (50 and 100 μg/mL) treatment significantly reduced the expression levels of all these genes, while the mRNA expression levels of Fabp4 and Acaca were reduced only by IHE treatment at 100 μg/mL.

### 3.4. IHE Inhibits Adipogenesis in MDI-Induced 3T3-L1 Cells, in Part, through the Regulation of MCE

**Figure 6:**
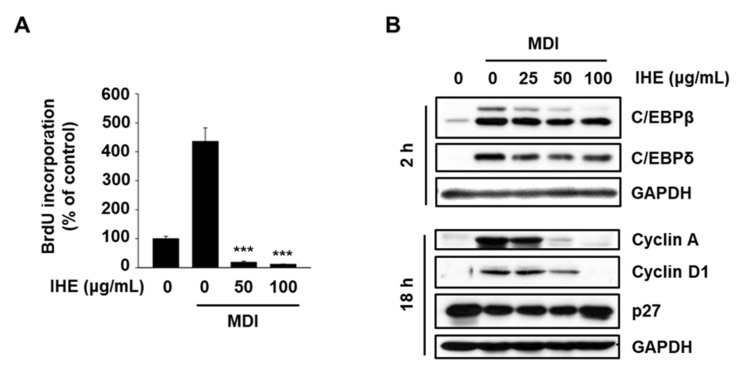
IHE inhibits MDI-induced MCE in growth-arrested 3T3-L1 cells. (**A**) Growth-arrested cells were treated with MDI for 18 h in the presence or absence of IHE, and BrdU incorporation was measured. (**B**) Growth-arrested cells were treated with MDI for 2 h or 18 h in the presence or absence of IHE, and whole cell lysates were analyzed by Western blot analysis; *** *p* < 0.005 vs. MDI alone.

In the previous study, we found that ISO regulates adipogenesis through inhibition of MCE [2], and therefore, we further investigated the effect of IHE on MCE during the adipogenic differentiation of 3T3-L1 cells. We first examined the effect of IHE on G1/S transition in MDI-treated 3T3-L1 cells using a bromodeoxyuridine (BrdU) incorporation assay. As seen in our previous study [2], BrdU incorporation (a marker of the S phase) reached a maximum at 18 h after MDI treatment, and therefore, growth-arrested 3T3-L1 cells were treated with IHE in the presence of MDI for 18 h. The results in Figure 6A showed that MDI treatment for 18 h dramatically induced G1/S transition, however, treatment with IHE at 50 and 100 μg/mL significantly inhibited the BrdU incorporation. Furthermore, treatment with IHE at 50 and 100 μg/mL markedly decreased protein expression of CEBPβ, cyclin A, and cyclin D1 while expression of CEBPδ and p27 was slightly decreased and increased, respectively (Figure 6B). These results suggest that IHE inhibited adipogenic differentiation of MDI-treated 3T3-L1 cells, in part, through the regulation of cell cycle regulatory proteins and key transcription factors modulating MCE.

## 4. Discussion

It is known that treatment with MDI causes growth-arrested 3T3-L1 cells to re-enter the cell cycle and undergo multiple mitoses in the early phase of adipogenic differentiation, followed by induction of the adipogenic genes [13,14]. The initiation of MCE involves the expression and activation of immediate early genes, such as transcription factors (CEBPβ and CEBPδ) and cell cycle regulators (p27, cyclin A, cyclin D1, etc.) which induce G1/S transition in 3T3-L1 cells, and many of these genes are known to be regulated by AMPK and Nur77 [1,2,15,16,17,18].

Increasing evidence has suggested that Nur77 is a new molecular target to regulate MCE during adipogenic differentiation of preadipocytes. Nur77 is induced rapidly by treatment with MDI in growth-arrested 3T3-L1 preadipocytes and initiates adipogenesis by inducing cell cycle re-entry and MCE [1,2]. Recently, we have also reported that transfection of siRNA for Nur77 (siNur77) significantly reduced G1/S transition, which is a prerequisite step for MCE during the initial phase of adipogenesis [2]. In addition, activation of AMPKα suppresses the MCE of preadipocytes in the early stage of adipogenic differentiation and decreases the expression of late adipogenic markers such as PPARγ and CEBPα [19,20]. Our recent study proposed the possibility of dual targeting of AMPKα and Nur77 as a potential antiadipogenic mechanism and confirmed that ISO, a sesquiterpene lactone found in *Inula helenium* roots, simultaneously inhibits Nur77 and activates AMPKα, significantly inhibiting adipogenesis in vitro and showing an anti-obesity effect in vivo [2].

In the present study, we first confirmed ALA, a positional isomer of ISO, also simultaneously targets Nur77 and AMPKα and inhibits adipogenesis of MDI-treated 3T3-L1 cells (Figure 1). In addition, in agreement with our hypothesis, IHE, which contains ISO and ALA as the major marker compounds, inhibited Nur77 and activated AMPKα (Figure 3), and suppressed adipogenesis of MDI-treated 3T3-L1 cells (Figure 4 and Figure 5), in part, through inhibition of MCE (Figure 6). The 70% ethanol extract of IH, which contains a larger amount of ISO and ALA as compared to the 30% ethanol extract, inhibited adipogenic differentiation of 3T3-L1 cells more effectively (data not shown), suggesting that these two compounds may contribute to the IHE-mediated inhibition of adipogenesis. IHE-mediated, MCE-inhibitory, and antiadipogenic effects were accompanied by decreasing expression of cyclin A and cyclin D1 in differentiating 3T3-L1 cells (Figure 6).

We have previously shown that ISO treatment during early time points (0–48 h after MDI treatment) exerts significant inhibitory effects on MDI-induced adipogenic differentiation of 3T3-L1 cells [2]. To investigate the IHE-mediated antiadipogenic mechanisms, we subsequently sought to determine which stage of differentiation is mainly affected by IHE treatment. As expected, IHE treatment during the early time point (treatment plans 3 and 4) showed significant inhibitory effects on the adipogenesis of MDI-treated 3T3-L1 cells (Supplementary Figure S2). However, unlike our hypothesis, treatment with IHE during differentiation days 2–6 (treatment plan 6) and days 4–6 (treatment plan 7) also showed significant antiadipogenic effects which were comparable to those observed in cells treated with IHE during differentiation days 0–2 (treatment plan 3) and days 0–4 (treatment plan 4), suggesting that the inhibitory effect of IHE, unlike ISO, is not limited to the early stage of adipogenesis. These results led us to examine the antiadipogenic mechanism of ALA and found that ALA affects not only the early stages of differentiation but also intermediate and late stages (Supplementary Figure S3). These results suggest that IHE-mediated antiadipogenic effect is due to the combined activity of ISO and ALA, and the mechanisms involved in the ALA- and IHE-mediated regulation of intermediate and terminal stages of adipogenesis are currently under investigation.

## 5. Conclusions

In conclusion, the present study shows that IHE and two IHE-derived sesquiterpene lactones, ALA and ISO, represent a new class of mechanism-based antiadipogenic agents that act, in part, via simultaneous regulation of AMPKα and Nur77. The data in the present study also suggest that IHE is a potential treatment option for obesity and obesity-related health problems. Our ongoing studies are focused on discovering other natural antiadipogenic agents which act through the dual targeting AMPKα and Nur77.

## Data Availability

Not applicable.

## Supplementary Materials

The following supporting information can be downloaded at: https://www.mdpi.com/article/10.3390/molecules27154765/s1, Figure S1: Effect of ALA and ISO on cell viability in MDI-treated 3T3-L1 cells; Figure S2: Effect of IHE on different stages of adipogenic differentiation in MDI-treated 3T3-L1 cells; Figure S3: Effect of ALA on different stages of adipogenic differentiation in MDI-treated 3T3-L1 cells; Table S1: Sequence of the primers used for qPCR.

## Sample Availability

Samples of the extract are available from the corresponding author.
