# Supplementary material for: Elecampane (Inula helenium) Root Extract and Its Major Sesquiterpene Lactone, Alantolactone, Inhibit Adipogenesis of 3T3-L1 Preadipocytes
Source: Molecules. 2022 Jul 25;27(15):4765. doi: 10.3390/molecules27154765 (PMC9332862; doi:10.3390/molecules27154765)
Supplement: Supplementary file 1 [file molecules-27-04765-s001.zip › molecules-1785808-supplementary.pdf]

Supplemental Materials

Table S1. Sequence of the primers used for qPCR.

Target gene[1)]	Primer sequence	
Tbp	Sense	5`-GTGAAGGGTACAAGGGGGTG-3`
	Antisense	5`-ACATCTCAGCAACCCACACA-3`
Pparγ	Sense	5`-GGAGCCTAAGTTTGAGTTTGCTGTG-3`
	Antisense	5`-TGCAGCAGGTTGTCTTGGATG-3`
Cebpα	Sense	5’-TTGAAGCACAATCGATCCATCC-3’
	Antisense	5’-GCACACTGCCATTGCACAAG-3’
Fabp4	Sense	5’-TGGGAACCTGGAAGCTTGTCTC-3’
	Antisense	5’-GAATTCCACGCCCAGTTTGA-3’
Fas	Sense	5’-AGCACTGCCTTCGGTTCAGTC-3’
	Antisense	5’-AAGAGCTGTGGAGGCCACTTG-3’
Scd-1	Sense	5’-CAGCCGAGCCTTGTAAGTTC-3’
	Antisense	5’-GCTCTACACCTGCCTCTTCG-3’
Acaca	Sense	5’-GAAGCCACAGTGAAATCTCG-3’
	Antisense	5’-GATGGTTTGGCCTTTCACAT-3’
Perilipin	Sense	5’-GATGAGAGCCATGACGACCAGA-3’
	Antisense	5’-TGTGTACCACACCACCCAGGA-3’

[1)]Tbp; TATA-binding protein, Pparγ; Peroxisome proliferator-activated receptor γ, C/ebpα; CCAT/enhancer binding protein alpha, Fabp4; Fatty acid binding protein 4, Fas; Fatty acid synthase, Acaca; Acetyl-CoA carboxylase alpha, Scd-1; Stearoyl-CoA desaturase-1.

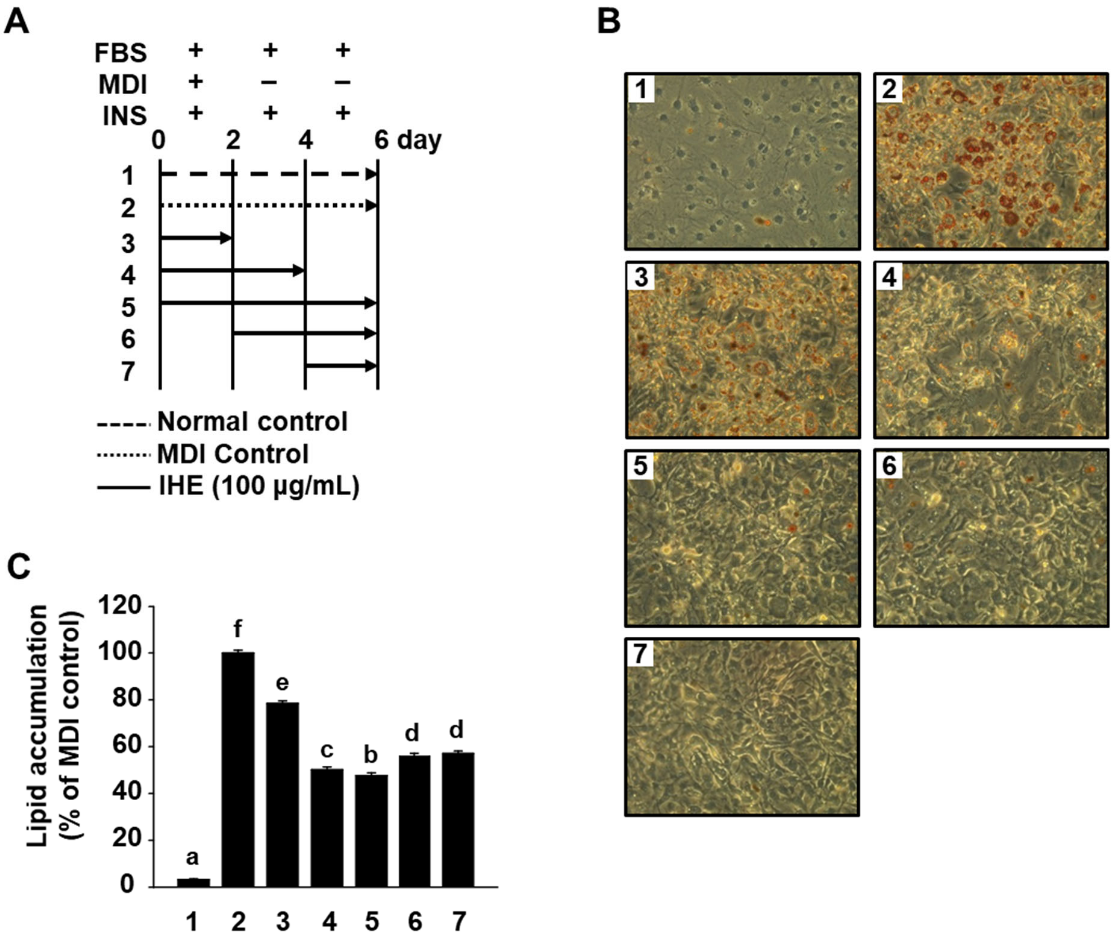

Figure S1. Effect of IHE on different stages of adipogenic differentiation in MDI-treated 3T3-L1 cells. A, Schematic model showing IHE treatment during adipogenic differentiation of 3T3-L1 cells. The arrows indicate the duration of IHE (100 µg/mL) treatment. B and C, Cells were stained with Oil Red O at 6 days after the induction of differentiation, and lipid accumulation was measured, as described in the Materials and Methods. The results are presented as means ± SEM (n≥3), and different letters are significantly different at $P < 0.05$ by Duncan's multiple range test.

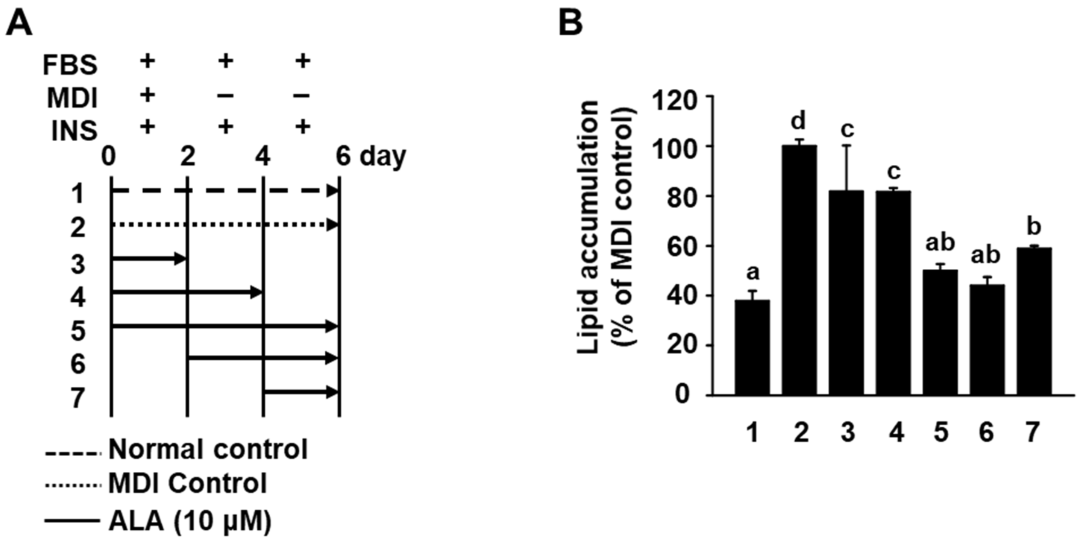

Figure S2. Effect of ALA on different stages of adipogenic differentiation in MDI-treated 3T3-L1 cells. A, Schematic model showing IHE treatment during adipogenic differentiation of 3T3-L1 cells. The arrows indicate the duration of ALA (10 μM) treatment. B and C, Cells were stained with Oil Red O at 6 days after the induction of differentiation, and lipid accumulation was measured, as described in the Materials and Methods. The results are presented as means ± SEM (n≥3), and different letters are significantly different at $P < 0.05$ by Duncan's multiple range test.
